# Electrophysiological and sick sinus syndrome effects of Remdesivir challenge in guinea-pig hearts

**DOI:** 10.3389/fphys.2024.1436727

**Published:** 2024-08-13

**Authors:** Shuang Li, Liang Yue, Yulong Xie, Henggui Zhang

**Affiliations:** ^1^ Key Laboratory of Medical Electrophysiology, Ministry of Education and Medical Electrophysiological Key Laboratory of Sichuan Province, (Collaborative Innovation Center for Prevention of Cardiovascular Diseases), Institute of Cardiovascular Research, Southwest Medical University, Luzhou, China; ^2^ Biological Physics Group, Department of Physics and Astronomy, The University of Manchester, Manchester, United Kingdom; ^3^ Beijing Institute of Artificial Intelligence, Beijing, China

**Keywords:** Remdesivir, cardiac electrophysiology, sick sinus syndrome, patch clamp, COVID-19

## Abstract

Remdesivir (RDV) is the first drug approved by the FDA for clinical treatment of hospitalized patients infected with COVID-19 because it has been shown to have good antiviral activity against a variety of viruses, including Arenaviridae and Coronaviridae viral families. However, it has been reported that its clinical treatment leads to the symptoms of sick sinus syndrome such as sinus bradycardia, conduction block, and sinus arrest, but the electrophysiological mechanism of its specific cardiac adverse events is still unclear. We report complementary, experimental, studies of its electrophysiological effects. In wireless cardiac telemetry experiments *in vivo* and electrocardiographic studies in *ex vivo* cardiac preparations, RDV significantly caused sinus bradycardia, sinus atrial block, and prolongation of the QT interval in guinea pigs. Dose-dependent effects of RDV on the electrical activities of sinoatrial node (SA node) preparations of guinea pigs were characterised by multielectrode, optical RH237 voltage mapping. These revealed reversibly reduced sinoatrial conduction time (SACT), increased AP durations (APDs), and decreased the pacemaking rate of the SA node. Patch-clamp experiments showed that RDV significantly inhibited the I_f_ current of HCN4 channels, resulting in a significant decrease in the spontaneous firing rate of SA node cells, which may underlie the development of sick sinus node syndrome. In addition, RDV significantly inhibits I_Kr_ currents in hERG channels, leading to prolongation of the QT interval and playing a role in bradycardia. Therefore, these findings provide insights into the understanding the bradycardia effect of RDV, which may be used as basic theoretical guidance for the intervention of its adverse events, and prompt safety investigations of RDV’s cardiac safety in the future.

## 1 Introduction

Remdesivir (RDV) is an adenosine triphosphate analogue first synthetically developed as a candidate for the treatment of hepatitis C virus and first described in the literature as a potential treatment for Ebola in 2016 (Warren et al., 2016). The mechanism of action of RDV suggests a broad antiviral activity spectrum. To date, *in vitro* studies have demonstrated its efficacy against multiple viral families including Filoviridae, Paramyxoviridae, Arenaviridae, Flaviviridae, Pneumoviridae, and Coronaviridae (Malin et al., 2020). Initial observations of RDV’s efficacy against the Coronaviridae family in 2017 sparked significant interest in its potential as a treatment for COVID-19 (Sheahan et al., 2017). However, concerns have been raised about its clinical safety in treating COVID-19. Nucleosides have long been associated with cardiovascular side effects, altering cardiomyocyte action potentials and potentially leading to adverse cardiac effects such as conduction block, atrial fibrillation, and ventricular fibrillation (Ahmad et al., 2015; Antonioli et al., 2023). Additionally, *in vitro* experiments have demonstrated that RDV can cause mitochondrial damage in cardiomyocytes (Bjork and Wallace, 2021; Kwok et al., 2022).

As COVID-19 escalates into an unparalleled global public health crisis, there is an urgent demand for efficacious drugs to treat clinically severe cases (Jeong et al., 2023). Research indicates that RDV can target the non-specific chain terminator of RdRp (RNA-dependent RNA polymerase) in SARS-CoV-2, as well as its related strains SARS-CoV and MERS-CoV, thereby exhibiting antiviral effects (De Wit et al., 2020). And studies have demonstrated clinical benefits of RDV treatment in rhesus monkeys infected with SARS-CoV-2 (Williamson et al., 2020). Consequently, RDV became the first drug to receive emergency authorization from the U.S. FDA for the treatment of hospitalized patients with COVID-19 under these conditions. Among 2,186 U.S. adult patients with laboratory-confirmed SARS-CoV-2 infection, RDV showed potential benefits for COVID-19 treatment (Rivera et al., 2020), and it also demonstrated a reduction in recovery time for hospitalized COVID-19 patients with lower respiratory tract infections (Williamson et al., 2020). However, with the clinical use of RDV, a significant number of cardiac adverse reactions have been reported. In a prospective longitudinal study involving COVID-19 patients, those receiving RDV treatment had an overall incidence of bradycardia (heart rate < 60 bpm) of 27%, with 19% of them experiencing severe bradycardia (heart rate < 50 bpm) (Hajimoradi et al., 2023). Al-Jammali et al. (2022) conducted a comprehensive review of published literature and a meta-analysis study focusing on RDV’s impact on SARS-CoV-2 patients, particularly those who experienced arrhythmias post RDV administration. Their findings revealed an elevated risk of sinus bradycardia associated with RDV treatment compared to control groups. The meta-analysis indicated a bradycardia incidence of 34.07% in RDV-treated patients, notably higher than the control group’s 18.13% incidence. Furthermore, a joint study by the FDA and the Academy of Medical Toxicology reported that among 760 patients, 220 developed sinus bradycardia post RDV use, with 181 of these cardiac adverse reactions directly attributed to RDV. The observed cardiac adverse events included sinus bradycardia, prolonged QRS/QT interval, sinus arrest, and cardiac arrest (Devgun et al., 2023). In summary, RDV’s association with sick sinus syndrome is confirmed, yet the precise electrophysiological mechanism triggering adverse cardiac reactions remains unclear.

Currently, research on RDV primarily focuses on clinical observations, with limited studies on its mechanisms. In this study, we first utilized wireless telemetry electrocardiography and *ex vivo* cardiac electrophysiology to examine RDV’s impact on *in vivo* and *ex vivo* guinea pig cardiac electrophysiology, revealing consistent results at both animal and ion cardiac levels with known clinical observations of the drug. Subsequently, we studied RDV’s effects on sinoatrial node (SA node) tissue pacing, action potential conduction, and APD duration under sinus rhythm-driven conditions, which have proven increasingly valuable in elucidating mechanisms of cardiac adverse reactions and possess significance in drug safety assessments, providing insights not attainable solely from *in vitro* single-cell single-channel studies (Strauss et al., 2019). Finally, we investigated RDV’s influence on acutely isolated SA node cells and the pivotal pacemaker ion channel HCN4 channel, while also examining RDV’s impact on hERG channel I_Kr_ currents in HEK293 cells, elucidating the drug’s actions at cellular and ion channel levels. Therefore, through the investigation of RDV’s impact on guinea pig cardiac electrophysiology, we can elucidate the clinical mechanisms behind RDV-induced sick sinus node syndrome and its implications for drug safety.

## 2 Methods

The experiments included the following: (1) Telemetric electrocardiography in healthy guinea pigs to investigate RDV’s impact on guinea pig cardiac electrophysiology *in vivo*. (2) *Ex vivo* electrocardiography studies on RDV’s effects on guinea pig *ex vivo* cardiac electrophysiology under Langendorff perfusion, further complementing the *in vivo* electrocardiography results. (3) Optical mapping and electrocardiographic techniques to study RDV’s effects on pacemaking signals, action potential conduction and duration in acutely isolated SA node tissues. (4) Patch clamp techniques to investigate RDV’s effects on acute isolated guinea pig SA node cells and the key ion channels involved in pacemaking. In experiments, we ensured the consistency of variables such as animal weight, procedural methods, experimental conditions, and analytical techniques. By minimizing variability across different experimental protocols, we enhanced the reliability of our results. Finally, we analysed findings from various levels to evaluate the electrophysiological mechanisms by which the drug of RDV induces sick sinus syndrome.

### 2.1 Ethics and study approval

All experimental procedures were approved by the Institutional Animal Care and Use Committee of Southwest Medical University and complied with the Guide for the Care and Use of Laboratory Animals published by the National Institutes of Health. Healthy female guinea pigs weighing 250–350 g were provided by Chongqing Tengxin Huafu Experimental Animal Sales Co., Ltd. They were housed at the Animal Center of Southwest Medical University with *ad libitum* access to food and water. Animals were euthanized humanely by intraperitoneal injection of 3% pentobarbital sodium (50 mg/kg).

### 2.2 Wireless cardiac telemetry experiments

The guinea pigs were placed in an anesthesia chamber and anesthetized with 3%–4% isoflurane. Once adequately anesthetized, the guinea pigs were transferred to the surgical table in a prone position and maintained under anesthesia using a face mask. The placement of the wireless sensor on the animal’s back, at the surgical incision site, was determined based on the guinea pig’s body size and electrode length. After local disinfection, the skin was incised with scissors, and two subcutaneous tunnels were created using a blunt needle from the pre-placed sensor site to the lead position. The electrode lead was then threaded through the tunnels, and the electrode ring was sutured onto the subcutaneous fascia at the lead position. Following successful electrode placement, wound closure and disinfection were completed, and the guinea pigs were housed in individual cages for recovery. They were used for electrocardiogram experiments 1 week after surgery. A backpack was installed to facilitate real-time electrocardiogram recording, capturing baseline ECGs for 24 h before and after intraperitoneal administration of RDV. Throughout the experiment, the animals had *ad libitum* access to food and water. In the *in vivo* electrocardiogram experiment, following clinical drug absorption patterns and treatment regimens, intraperitoneal drug administration was used to study drug-induced cardiac adverse reactions. The dosing regimen for this animal experiment spanned 5 days, with recordings taken for 24 h before drug administration, 24 h after the first dose, 24 h after the fifth dose, and 24 h after a recovery period of 3 days post-treatment cessation.

### 2.3 Langendorff-perfused isolated hearts

Guinea pigs (female; 250–350 g) were humanely killed by intraperitoneal injection with pentobarbital sodium (50 mg/kg). Hearts rapidly excised after thoracotomy were mounted onto a Langendorff perfusion system and perfused with a modified Tyrode’s solution (119 NaCl, 25 NaHCO_3_, 4 KCl, 1.2 KH_2_PO_4_, 1 MgCl_2_, 1.8 CaCl_2_·2H_2_O, and 10 D-glucose (in mM) equilibrated with 5% CO_2_ and 95% O_2_) with the flow rate of 8 mL/min at 37°C. Hearts were perfused and monitored for stability for 20 min before experimental procedures commenced.

### 2.4 Measurement of heart rate in Langendorff perfusion-maintained *ex vivo* hearts

ECG was recorded via two silver electrodes attached to the ventricular apex and to the aortic cannula. An equilibration period of approximately 20 min was allowed to ensure stable ECG recordings. The hearts were then successively perfused with RDV at concentrations of 0.1, 0.3, 1, 3 and 10 μM for 15 min with intervening washout periods of 15 min. It should be noted that the effect of RDV on heart rate was almost reversed during the washout period.

### 2.5 Optical mapping of sinoatrial node region

After the Langendorff-perfused hearts reached steady state, contraction artifacts were mimimized using blebbistatin (10 μM). RH237 (1 μg/mL) were perfused to enable membrane potential at 37°C. The hearts were dissected to prepare preparations containing left and right atria in 37°C bath. ECG electrodes were placed on the right and left auricles to record the pacemaking rate of the SA node tissue. An equilibration period of approximately 10 min was allowed to ensure the stable pacemaking recordings. RH237 (1 μg/mL) were perfused to enable membrane potential again. Two 530 nm LEDs were used to illuminate the heart after their emissions were bandpass filtered (wavelengths 530 ± 20 nm) to minimize stray excitation light reaching the dyes. The fluorescence light was passed through a 550 nm long-pass filter and then a dichroic mirror with a cutoff of 638 nm. Fluorescence light with wavelengths above 638 nm was passed through a 700 nm long-pass filter and then imaged by the camera for recording voltage signals. The cameras of the optical mapping system, LED lights, and ECG recording were simultaneously driven using OMapRecord 4.0 software. For the analysis of optical mapping signals and generation of isochronal maps, data were semiautomatically processed using Electromap software. Furthermore, the interval between the initial pacing center point of the sinoatrial node and crista terminalis, during which the action potential travels, was designated as the sinus atrial conduction time (SACT).

### 2.6 SA node cell preparation

Single SA node cells were isolated using an enzymatic dispersion procedure similar to that described previously (Guo et al., 1997; Kojima et al., 2012). Briefly, guinea-pigs were deeply anaesthetized with sodium pentobarbital (i.p., 120 mg kg^−1^), and then the chest cavity was opened under artificial respiration. The ascending aorta was cannulated *in situ* and the heart was then excised and retrogradely perfused via the aortic cannula on a Langendorff apparatus at 37°C, initially for 4 min with normal Tyrode solution containing (in mM) 140 NaCl, 5.4 KCl, 1.8 CaCl_2_, 0.5 MgCl_2_, 0.33 NaH_2_PO_4_, 5.5 glucose and 5 HEPES (pH adjusted to 7.4 with NaOH), and then for 4 min with nominally Ca^2+^-free Tyrode solution (made by simply omitting CaCl_2_ from the normal Tyrode solution). This was followed by 8–12 min of perfusion with nominally Ca^2+^-free Tyrode solution containing 0.4 mg mL^−1^ collagenase (Wako Pure Chemical Industries, Osaka, Japan). All these solutions were oxygenated with 100% O_2_. The digested heart was then removed from the Langendorff apparatus, and the SA node region, bordered by the crista terminalis, the intra-atrial septum, and superior and inferior vena cava, was dissected out and cut perpendicular to the crista terminalis into small strips measuring about 0.5 mm in width. These SA node tissue strips were further digested at 37°C for 20 min with nominally Ca^2+^-free Tyrode solution containing 1.0 mg mL^−1^ collagenase and 0.1 mg mL^−1^ elastase. Finally, the enzymatically digested SA node strips were gently agitated in a high-K^+^, low-Cl^−^ Kraftbrühe (KB) solution, containing (in mM) 70 K-glutamate, 30 KCl, 10 KH_2_PO_4_, 1 MgCl_2_, 20 taurine, 0.3 EGTA, 10 glucose and 10 HEPES (pH adjusted to 7.2 with KOH) (Isenberg and Klockner, 1982). The isolated cells thus obtained were then stored at 4°C in the KB solution for experimental use within 8 h.

### 2.7 Whole-cell patch-clamp recordings

Perforated and conventional (ruptured) whole-cell patch-clamp techniques were used to record the spontaneous action potentials, I_f_ currents and I_Kr_ currents in the current- and voltage-clamp modes respectively (Hamill et al., 1981; Horn and Marty, 1988). An EPC-10 patch-clamp amplifier was used for these recordings. The fire-polished patch electrodes had resistance of 3.0–6.0 MΩ when filled with the pipette solution. In experiments, the cells adhered to the Round Coverslip were transferred to the recording chamber (volume of 0.5 mL) mounted on the stage of an inverted microscope. The chamber was perfused with standard Tyrode’s solution at a temperature of 35°C–37°C. The cells included cultured HEK293 cells stably transfected with hERG channels and acutely isolated SA node cells. The SA node cells dissociated from the entire SA node region comprised morphologically heterogeneous cells, such as spindle- and spider-shaped cells. Because spindle-shaped cells closely resemble the primary pacemaker cells in the SA node (Belardinelli et al., 1988), such cells displaying regular contraction were selected for the present experiments to assess the effect of RDV on SA node electrical activity.

Spontaneous action potentials were recorded in normal Tyrode solution with a pipette solution containing (in mM) 70 potassium aspartate, 50 KCl, 10 KH_2_PO_4_, 1 MgSO_4_ and 5 HEPES (pH adjusted to 7.2 with KOH). Amphotericin B (150 μg·mL^−^) was added to the pipette solution just before use and measurements were started 5–10 min after giga-seal formation. I_f_ was recorded in normal Tyrode solution with a K^+^-rich pipette solution containing (in mM) 70 potassium aspartate, 50 KCl, 10 KH_2_PO_4_, 1 MgSO_4_, 5 ATP, 0.1 GTP, 5 EGTA, 1.2 CaCl_2_ and 5 HEPES (pH adjusted to 7.2 with KOH). I_f_ was activated by hyperpolarizing voltage-clamp steps applied from a holding potential of −40 mV to test potentials in 10 mV decrements from −40 to −130 mV for a duration of 2.5 s, followed by a return to −40 mV. I_Kr_ was recorded in normal Tyrode solution with a pipette solution containing (in mM): KOH 110, KCl 40, K_2_ATP 5, MgCl_2_ 5, EGTA 5, HEPES 10 (pH was adjusted to 7.2 by KOH). The hERG current was assessed using the patch-clamp technique with the membrane potential clamped at −80 mV. Depolarizing voltage steps were applied in 10 mV increments from −60 mV to +60 mV for a duration of 3 s, followed by a return to −50 mV.

### 2.8 Statistical analysis

*In vivo* electrocardiographic data were analyzed using LabChart 7.0 (ADInstruments). *Ex vivo* cardiac electrograms were analyzed using Spike 2 software (CED). Data from optogenetic experiments were analyzed using ElectroMap, with action potential durations (APDs) measured as the time required for 50% and 80% repolarization. Raw current data from patch clamp experiments were analyzed using Clampfit 10.2 (Molecular Devices, United States). All data are presented as mean ± standard deviation (SD). Comparisons between the two groups were performed using the unpaired Student’s t-test. One-way ANOVA, followed by *post hoc* tests, were performed to compare multiple groups using GraphPad Prism8 software (GraphPad Software, San Diego, CA, United States). Statistical significance was set at *p* < 0.05. In the figures, the designations for *p* values are *p < 0.05, **p < 0.01 and ***p < 0.001 respectively.

## 3 Results

### 3.1 Wireless cardiac telemetry experiments in guinea pigs

Firstly, we investigated the impact of RDV on *in vivo* guinea pig cardiac electrophysiology using wireless telemetry electrocardiography. Referencing clinical RDV treatment concentrations and regimens for COVID-19 patients, we administered RDV via intraperitoneal injection to guinea pigs (n = 7) for 5 days, with an initial dose of 23.6 mg/kg on day 1 and 11.8 mg/kg on days 2–5. We recorded the guinea pigs’ normal cardiac electrophysiology before administration, as well as on the first and fifth days of administration, and the third day after the last dose. In guinea pigs, the normal heart rate in control conditions ranges from 200 to 360 beats per minute (bpm). According to Haq et al. (2022), young healthy guinea pigs exhibit a heart rate not lower than 230 bpm. Therefore, heart rates below 200 bpm can be classified as bradycardia.

**FIGURE 1:**
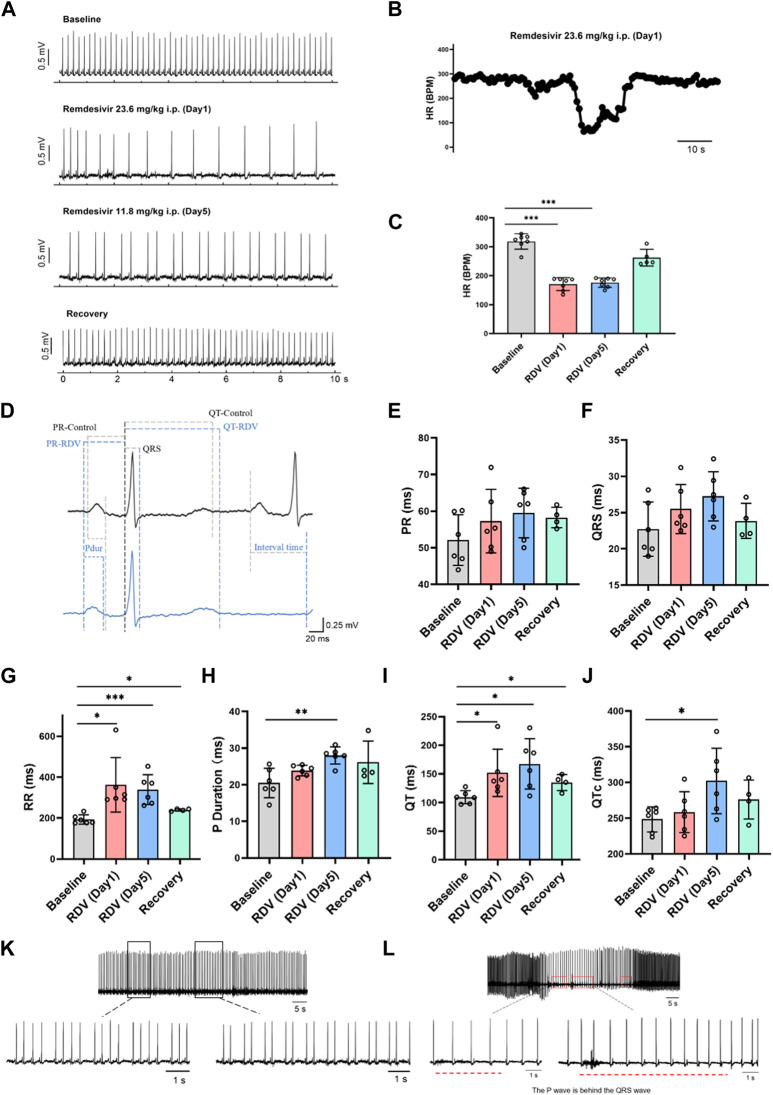
Effect of RDV on electrocardiography in guinea pigs. **(A)** Representative electrocardiogram of guinea pigs before and after RDV administration and recovery. **(B)** Representative segment showing the severe decrease in heart rate of guinea pigs induced by RDV. **(C)** Heart rate statistics of guinea pigs before and after RDV administration (n = 7). **(D)** Representative electrocardiogram of guinea pigs before and after RDV administration. **(E)** Statistical chart of PR interval. **(F)** Statistical chart of QRS. **(G)** Statistical chart of RR interval. **(H)** Statistical chart of P duration. **(I)** Statistical chart of QT interval. **(J)** Statistical chart of QTc (n = 6). **(K)** RDV causes second-degree type I and second-degree type II cardiac conduction block. **(L)** RDV induces junctional escape rhythm.

Figure 1A shows that compared to baseline, RDV administration significantly reduced heart rates in guinea pigs on the first and fifth days, with heart rates returning to normal after cessation of RDV. Figure 1B illustrates that RDV led to severe sinus bradycardia in guinea pigs, with the lowest heart rate dropping to 60 BPM. Additionally, we conducted statistical analysis on guinea pig heart rates 24 h before and after administration. As shown in Figure 1C, as compared to controls, intraperitoneal RDV significantly decreased sinus heart rates on the first and fifth days. Additionally, two guinea pigs experienced sudden cardiac arrest and death following the conclusion of the administration period. Therefore, the *in vivo* cardiac electrophysiology results in guinea pigs suggest that RDV significantly inhibits sinus node pacing function, leading to a marked decrease in sinus heart rate. It may also cause sinus node organic lesions, preventing recovery to the optimal state before administration and potentially resulting in sudden cardiac arrest after discontinuation.

Subsequently, we analyzed electrocardiographic parameters (Figure 1D), revealing that RDV had no significant effect on the PR interval (Figure 1E) or QRS duration (Figure 1F). However, RDV notably prolonged the RR interval (Figure 1G), P duration after the fifth day of administration (Figure 1H), QT interval (Figure 1I), and QTc (Figure 1J) after the fifth day of administration. Prolongation of the RR interval, P duration, and QT interval suggests that RDV not only affects sinus node pacing function but also influences sinus atrial conduction, with concomitant prolonged ventricular repolarization. Among these effects, RDV-induced suppression to SA node pacing function appears to be the primary factor contributing to sinus bradycardia.

Finally, we observed that RDV not only induced severe sinus bradycardia with arrhythmia but also elicited characteristic electrocardiographic responses representative of sick sinus syndrome. These included RDV-induced manifestations of marked second-degree type I and type II sinus atrioventricular (AV) block in guinea pigs (Figure 1K), further indicating severe sinus AV conduction block. Moreover, RDV also resulted in junctional escape rhythms in guinea pigs (Figure 1L), where P waves appeared after QRS complexes, underscoring RDV’s profound impact on SA node function, leading to sinus arrest and initiation of escape pacemaker activity. This pacemaker first activated the ventricles, then retrogradely activated the atria, while maintaining a bradycardic state throughout, suggesting RDV’s suppressive effect on cardiac pacing. In addition, some guinea pigs experienced profound bradycardia, culminating in sudden cardiac arrest.

In summary, these findings demonstrate that RDV disrupts SA node pacing function, leading to sinus atrioventricular conduction block, QT prolongation, resulting in severe bradycardia and arrhythmia, ultimately culminating in sick sinus syndrome.

### 3.2 Electrocardiographic studies in *ex vivo* cardiac preparations

Next, we excluded the influence of other factors such as neural and hormonal effects and investigated the impact of RDV on *ex vivo* guinea pig cardiac electrophysiology using Langendorff perfusion.

**FIGURE 2:**
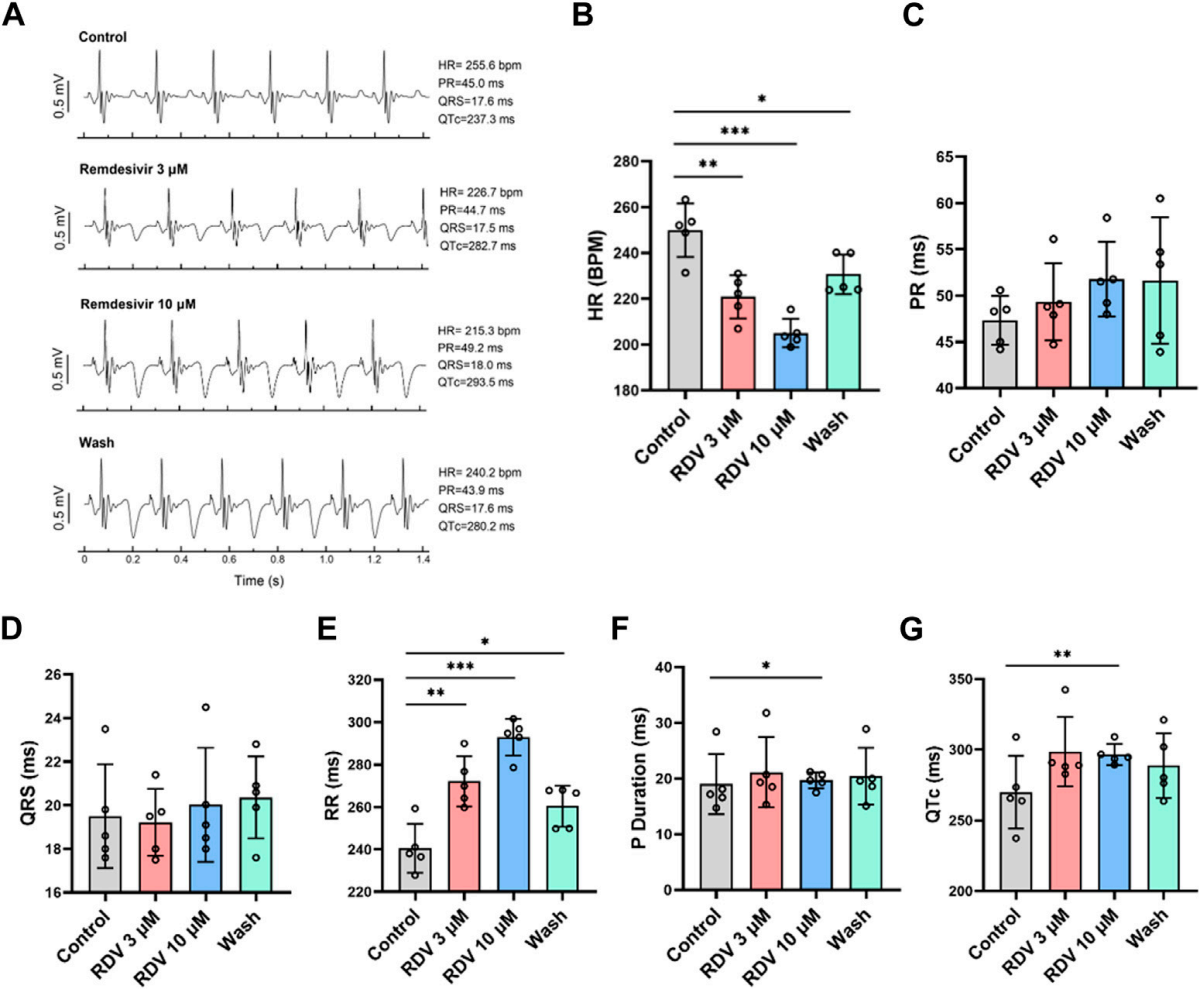
Effect of RDV on electrocardiography in Langendorff-perfused guinea pig isolated hearts. **(A)** Representative electrocardiograms of guinea pig isolated hearts before and after administration of 3 μM RDV, 10 μM RDV and after wash. **(B)** A statistical graph depicting the changes in heart rate of guinea pig isolated hearts before and after the administration of 3 μM and 10 μM RDV (n = 5). **(C)** Statistical chart of PR interval. **(D)** Statistical chart of QRS. **(E)** Statistical chart of RR interval. **(F)** Statistical chart of P duration. **(G)** Statistical chart of QTc (n = 5).

Administering various concentrations of RDV (0.1 μM, 0.3 μM, 1 μM, 3 μM, 10 μM) to *ex vivo* guinea pig hearts via Langendorff perfusion, we monitored electrocardiographic changes pre- and post-administration. Our results revealed that compared to the control, both 3 μM and 10 μM RDV significantly slowed *ex vivo* guinea pig heart rates, with recovery observed upon washout (Figures 2A,B). Conversely, lower concentrations of 0.1 μM, 0.3 μM, and 1 μM RDV showed no significant impact on *ex vivo* cardiac rhythm (Supplementary Table S1). Noticeably, there exist significant discrepancies in heart rates between *ex vivo* and *in vivo* guinea pig hearts, attributed to the inability of *ex vivo* experimental conditions to fully replicate the physiological state of the heart *in situ*, including the regulatory influences such as neural and hormonal inputs. Consequently, *ex vivo* cardiac conditions cannot precisely replicate the exact physiological status of the *in vivo* heart, thus producing inherent baseline differences between these two conditions (Wang et al., 2021).

Subsequently, we conducted further analysis of *ex vivo* guinea pig cardiac electrophysiological parameters. Our results indicated that different concentrations of RDV had no significant impact on PR interval (Figure 2C) or QRS duration (Figure 2D). However, 3 μM and 10 μM RDV significantly prolonged the RR interval (Figure 2E), with only 10 μM RDV causing significant prolongation of P duration (Figure 2F), and QTc (Figure 2G).

The consistent findings between *ex vivo* guinea pig cardiac electrophysiology and *in vivo* guinea pig cardiac electrophysiology indicate that RDV primarily inhibits SA node pacing function, leading to sinus atrioventricular conduction block, resulting in sick sinus syndrome, accompanied by a certain degree of QTc prolongation.

### 3.3 Optical mapping studies in *ex vivo* SA node preparations

**FIGURE 3:**
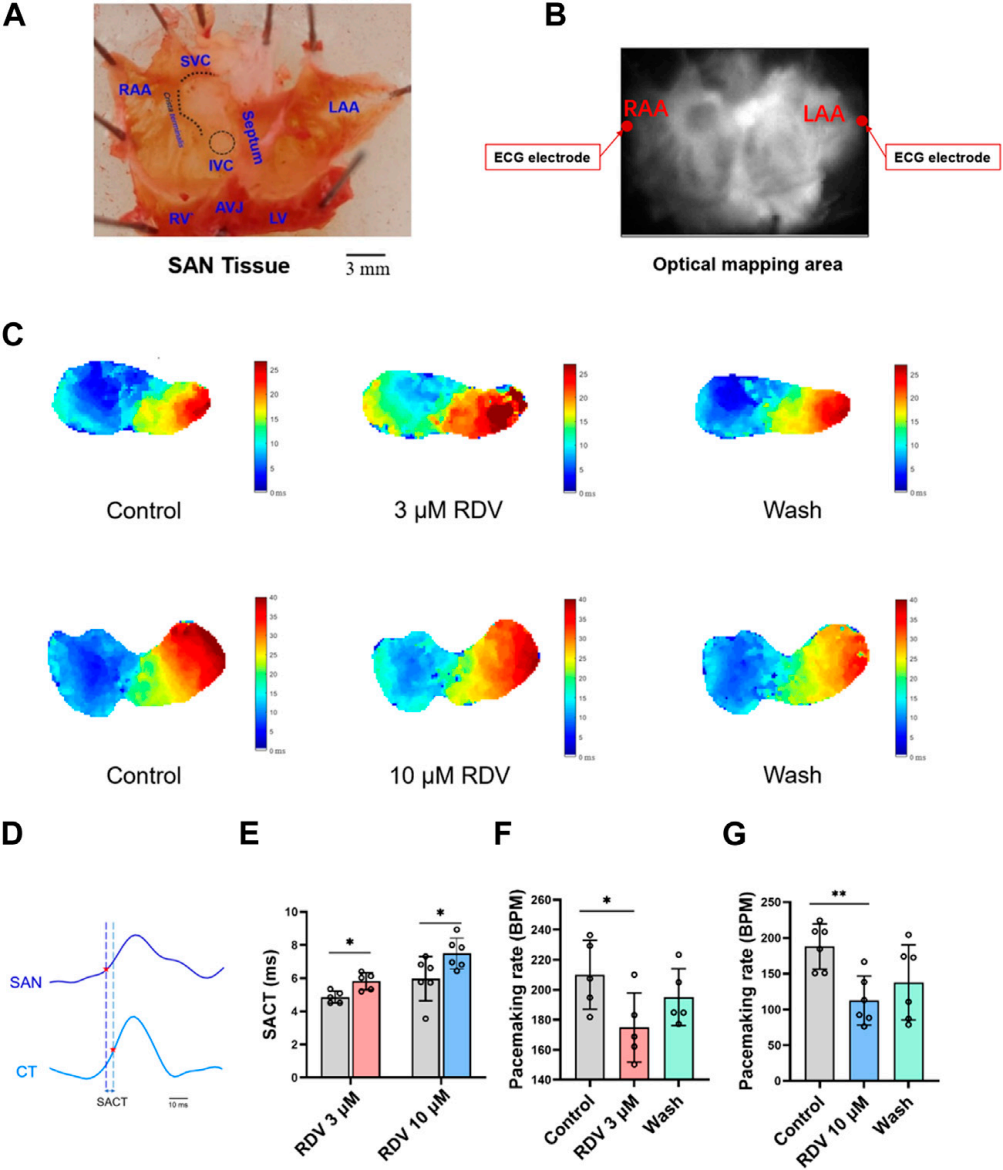
Effect of RDV on pacemaking rate and AP conduction in SA node preparations of guinea pig. **(A)** Schematic representation of the SA node (SAN) preparations after dissection. RAA, Right Atrial Appendage; LAA, Left Atrial Appendage; SVC, Superior Vena Cava; IVC, Inferior Vena Cava; RV, right ventricle; LV, Left Ventricle; AVJ, Artioventricular Junction. **(B)** Schematic of the optical mapping area and ECG recording configuration. **(C)** Representative isochronal conduction maps of SA node tissue before and after administration of 3 μM and 10 μM RDV. **(D)** Schematic of conduction time from the SA node site to the crista terminalis (SACT), CT, Crista terminalis. **(E)** Statistical graph of SACT after the administration of 3 μM (n = 5) and 10 μM (n = 6) of RDV. **(F)** A statistical graph depicting the changes in pacemaking rate of sinus node before and after the administration of 3 μM RDV (n = 5). **(G)** A statistical graph depicting the changes in pacemaking rate of sinus node before and after the administration of 10 μM RDV (n = 6).

Next, we employed optical mapping techniques to investigate the effects of RDV on acutely dissected guinea pig SA node tissue. The dissected tissue consisted of cardiac tissue from the left and right atria and atrial appendages, with the SA node located as a translucent structure between the superior and inferior vena cava in the right atrium and the crista terminalis (Figure 3A). Recording electrodes were placed on both sides of the left and right atrial appendages, and the optical mapping data acquisition area covered the entire left and right atria (Figure 3B).

**FIGURE 4:**
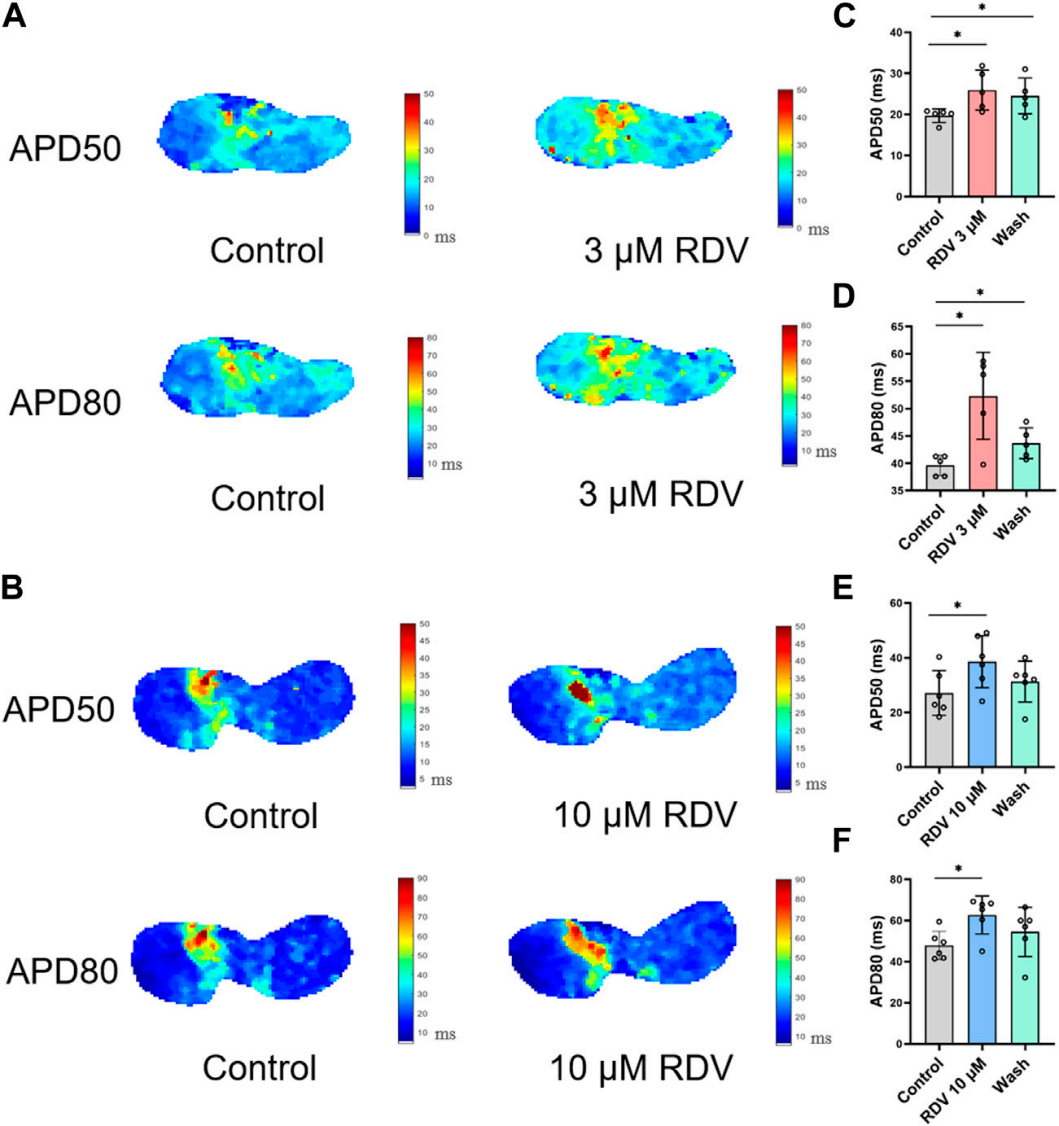
Effect of RDV on APD50 and APD80 in SA node tissue. **(A)** Effect of 3 μM RDV on APD50 and APD80 in SA node tissue. **(B)** Effect of 10 μM RDV on APD50 and APD80 in SA node tissue. **(C)** APD50 statistics after administration of 3 μM RDV (n = 5). **(D)** APD80 statistics after administration of 3 μM RDV (n = 5). **(E)** APD50 statistics after administration of 10 μM RDV (n = 6). **(F)** APD80 statistics after administration of 10 μM RDV (n = 6).

Figure 3C illustrates voltage mapping measurements of action potential initiation and conduction under sinus rhythm conditions. The results of sinoatrial conduction time (Figures 3D,E) indicated sinoatrial conduction block under the influence of 3 μM and 10 μM RDV, while SA node pacing rates (Figures 3F,G) showed a significant decrease, along with visible conduction heterogeneity and slowing (Figure 3C). Finally, optical action potential maps (Figures 4A–F) demonstrated that 3 μM and 10 μM RDV prolonged the duration of action potentials at 50% and 80% repolarization (APD50 and APD80) in SA node tissue.

### 3.4 Patch-clamp studies of spontaneous activity and HCN4 channel characteristics in SA node cells

**FIGURE 5:**
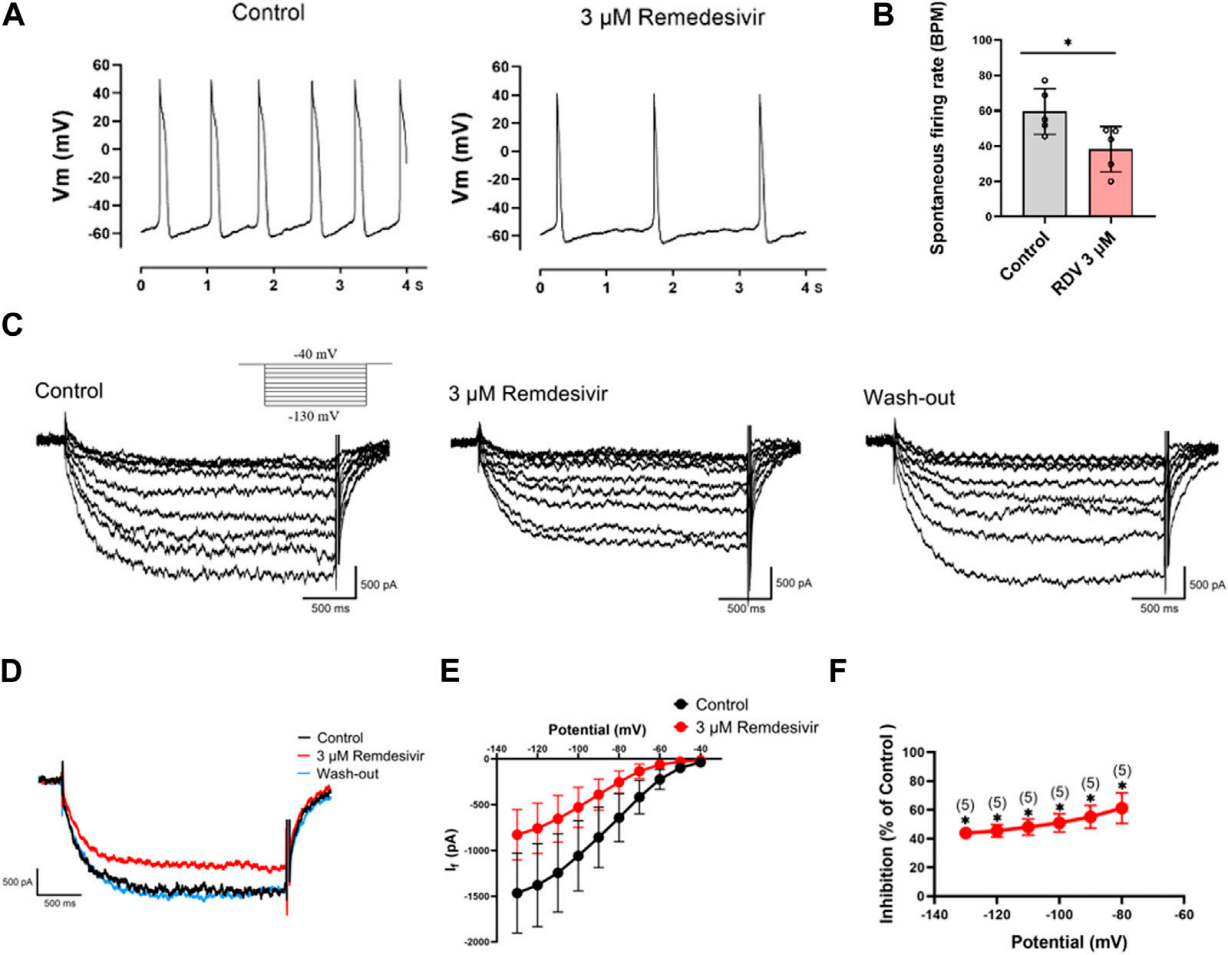
Effect of RDV on the pacemaking rate and HCN4 channel characteristics of SA node cells. **(A)** Representative plot of the effect of administration of 3 μM RDV on the Pacemaking rate of SA node cells. **(B)** Statistical plot of the effect of 3 μM RDV on SA node cell beating rate (n = 5). **(C)** Representative plots of I_f_ current changes before and after administration of 3 μM RDV. **(D)** Representative plots of the effect of 3 μM RDV on I_f_ current at a hyperpolarizing voltage of − 130 mV. **(E)** I-V curves of I_f_ current before and after 3 μM RDV administration (n = 5). **(F)** Suppression of current at hyperpolarizing voltage (n = 5).

Next, we acutely isolated guinea pig SA node cells and utilized patch-clamp techniques to investigate the effects of RDV on spontaneous action potentials and the key pacemaker ion channel HCN4 channel I_f_ current. The results revealed that 3 μM RDV significantly decreased the pacing rate of SA node cells (Figures 5A,B). Additionally, the original current traces of the HCN4 channel (Figure 5C) and current traces at −130 mV (Figure 5D) showed that 3 μM RDV inhibited the HCN4 channel, leading to a significant reduction in I_f_ current. Moreover, the inhibition rate of I_f_ current by 3 μM RDV exceeded 40% (Figures 5E,F). Therefore, our study has confirmed that RDV significantly suppresses the crucial pacemaker current I_f_ current in SA node cells, contributing to a decrease in the pacing rate of sinoatrial node cells.

### 3.5 Patch-clamp studies of hERG channel characteristics in HEK293 cells

**FIGURE 6:**
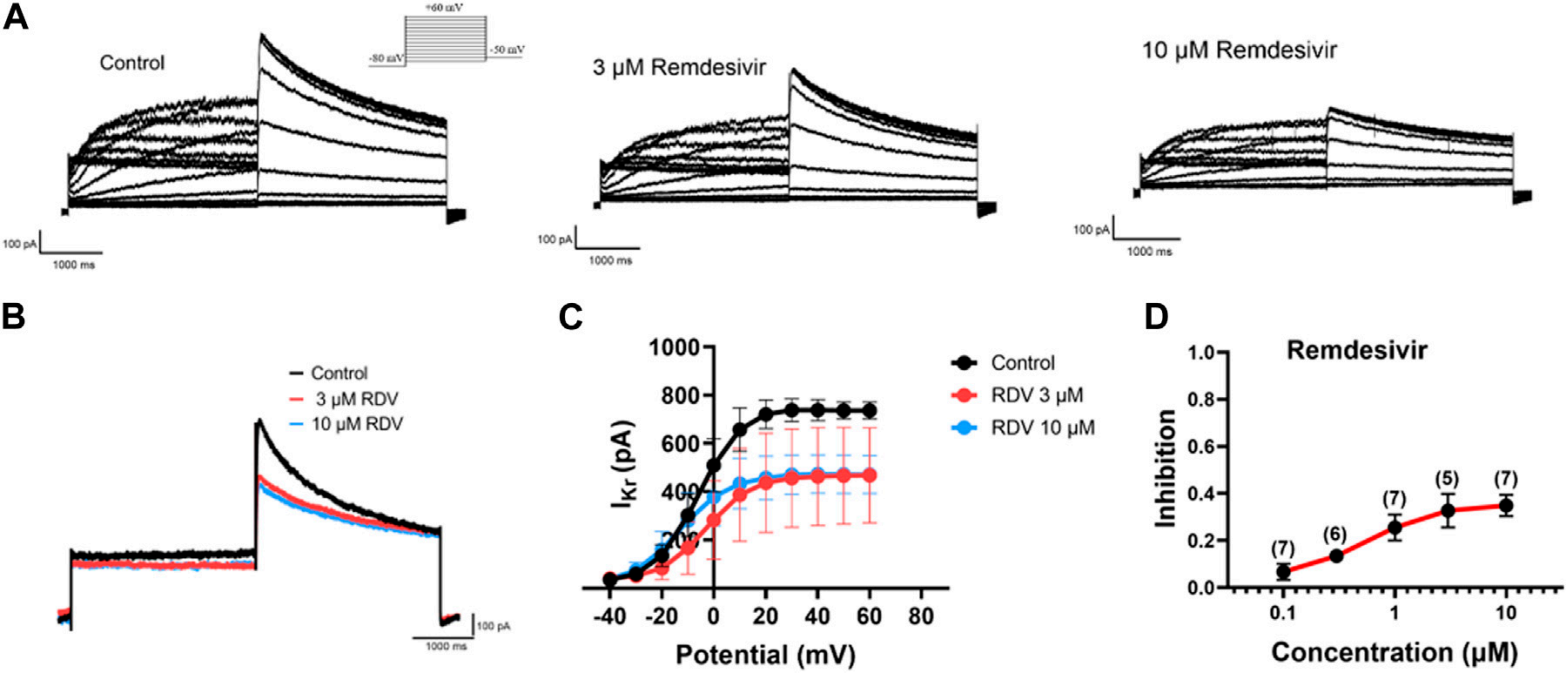
Effect of RDV on I_Kr_ current of hERG channels in HEK293. **(A)** Representative plots of I_Kr_ current changes before and after administration of 3 μM and 10 μM RDV. **(B)** Effect of administration of 3 μM RDV and 10 μM RDV on I_Kr_ current at 60 mV voltage. **(C)** The I-V curve graph of I_Kr_ current after the action of 3 μM (n = 5) and 10 μM RDV (n = 7). **(D)** Inhibition of hERG channels by different concentrations of RDV.

Finally, due to the observed QT prolongation in both clinical and our *in vivo* and *in vitro* electrocardiographic data, we further investigated the effects of RDV on hERG channel I_Kr_ current using patch-clamp techniques in HEK293 cells. The results demonstrated that both 3 μM and 10 μM RDV significantly inhibited the hERG channel current compared to the control group (Figures 6A,B). Moreover, the I-V curve analysis indicated that the inhibition of the hERG channel by RDV was voltage-dependent (Figure 6C). Additionally, as illustrated in Figure 6D, the inhibition of the hERG channel by RDV displayed concentration-dependence, with average inhibition rates of 32.7% and 34.9% for I_Kr_ current at 3 μM and 10 μM RDV, respectively. These findings suggest that RDV can lead to inhibition of the hERG channel, and the reduction in I_Kr_ current due to hERG channel inhibition can result in QT interval prolongation.

## 4 Discussion

This study investigated the effects of RDV on guinea pig hearts and isolated guinea pig heart electrophysiology under Langendorff perfusion to assess whether the obtained results align with clinical observations. Furthermore, we explored the impact of RDV on SA node tissue pacing, action potential signal propagation, pacing of SA node cells, and the HCN4 ion channel in acute isolated SA node cells, thereby further studying the electrophysiological mechanism of RDV-induced sick sinus syndrome at the tissue, cellular, and channel levels. Additionally, we examined the effects of RDV on hERG channel I_Kr_ current in HEK293 cells to confirm its association with clinical QT interval prolongation. This study represents the first comprehensive investigation into the cardiac electrophysiological effects of RDV, incorporating animal electrocardiography, *ex vivo* cardiac electrophysiology, SA node tissue pacing, action potential conduction, SA node cell pacing, and ion channel alterations. Finally, we correlate the observed cardiac electrophysiological responses induced by RDV with its potential to cause sick sinus syndrome clinically. The diagnosis of sick sinus syndrome, the latter of which is based on drug-induced bradycardia, conduction block, QT interval prolongation, and cardiac arrest, among other electrocardiographic abnormalities observed in *in vivo* electrocardiography, *ex vivo* cardiac electrophysiology, and sinoatrial node tissue conduction, involves symptoms such as sinus bradycardia, sinus arrest or pause, sinoatrial exit block, chronotropic incompetence, or bradycardia-tachycardia syndrome (Brignole et al., 2013; Kusumoto et al., 2019). The investigation into the electrophysiological mechanism of RDV-induced sick sinus syndrome stems from numerous clinical case reports of adverse cardiac events, including bradycardia, conduction blocks, QT interval prolongation, and cardiac arrest (Nabati and Parsaee, 2022; Nie et al., 2022; Wu et al., 2022; Ai et al., 2023; Devgun et al., 2023; Kanagala et al., 2023), highlighting the critical need for mechanistic research to elucidate the cardiac adverse effects of RDV in COVID-19 patients.

Our study yielded the following findings: Telemetry electrocardiography in guinea pigs revealed that RDV induced sick sinus syndrome symptoms, including sinus bradycardia, sinoatrial conduction block, junctional escape rhythm, and sinus arrest, as well as long QT syndrome. Prolongation of P duration also indicated RDV-induced sinoatrial conduction block, consistent with clinical observations. Additionally, electrocardiographic parameters showed RDV-induced QT interval prolongation, aligning with clinical findings. Moreover, *ex vivo* electrocardiography results similarly demonstrated that μM RDV caused bradycardia, sinoatrial conduction block, and QT interval prolongation, with heart rate recovery upon washout, consistent with our *in vivo* and clinical findings, thus providing further evidence of the adverse cardiac effects of RDV. Furthermore, we employed optical mapping techniques for the first time to study the effects of RDV on action potential conduction and duration at the sinoatrial node tissue level. Tissue-level results indicated that RDV inhibited sinoatrial node pacing and prolonged action potential conduction time and duration, further elucidating the reasons for RDV-induced sick sinus syndrome at the *ex vivo* cardiac and animal levels. Lastly, we investigated for the first time at the acute isolated sinoatrial node cell level the effects of RDV on native cell pacing and the key pacing ion channel HCN4 channel, revealing significant inhibition of sinoatrial node cell pacing by RDV and significant inhibition of the key pacing ion channel HCN4 channel’s I_f_ current. Additionally, RDV significantly inhibited hERG channel I_Kr_ current. Thus, further ion channel and cellular evidence confirmed that RDV causes a decrease in sinoatrial node cell pacing by inhibiting the key pacing current I_f_, leading to decreased sinoatrial node tissue pacing accompanied by sinoatrial conduction tissue and action potential duration prolongation, ultimately resulting in symptoms of sick sinus syndrome such as sinus bradycardia, sinoatrial conduction block, junctional escape rhythm, and sinus arrest. Furthermore, RDV significantly inhibits hERG channel I_Kr_ current, leading to QT prolongation, presenting another cause of bradycardia.

Previous studies on RDV have primarily focused on clinical cases and a few *in vitro* investigations. One pediatric case with severe acute COVID-19 showed sinus bradycardia during RDV treatment (Al-Jammali et al., 2022). In a prospective longitudinal study of COVID-19 patients, the overall incidence of bradycardia (heart rate < 60 bpm) was 27% among those treated with RDV, with 19% of them experiencing severe bradycardia (heart rate < 50 bpm) (Hajimoradi et al., 2023). Furthermore, a study by the US FDA and the Medical Toxicology Academy confirmed RDV-induced adverse cardiac reactions including sinus bradycardia, QRS/QT interval prolongation, sinus arrest, and cardiac arrest (Devgun et al., 2023). These previous findings underscore the cardiac adverse effects of RDV in clinical settings. Our *in vivo* and *ex vivo* electrocardiography results further demonstrate that RDV can induce sick sinus syndrome and prolong QT intervals.

Previous studies by Wolfes et al. (2024) and Pilote et al. (2022) have also demonstrated that RDV can cause a decrease in heart rate in isolated hearts, accompanied by QT interval prolongation and shortened ventricular APD90. Note that their research focused on the ventricular region of the heart, specifically on QT interval prolongation. However, based on our *in vivo* electrocardiographic and *ex vivo* heart findings in guinea pigs, QT interval prolongation is not the primary cause of RDV-induced cardiac adverse effects. Instead, dysfunction of the sinoatrial node (SAN) is the major factor. Therefore, our study primarily investigates the conduction and APD of excitation in the SA node region, revealing that RDV leads to reduced SAN pacing, sinoatrial conduction block, and APD prolongation. Moreover, this study represents the first to utilize optical mapping techniques to investigate RDV’s effects on action potential conduction and duration at the sinoatrial node tissue level, providing valuable insights into the mechanisms of cardiac adverse reactions and enhancing drug safety assessment by offering experimental insights not achievable solely through *in vitro* single-cell single-channel studies (Guo et al., 2009). These results further elucidate the pathogenesis of RDV-induced sick sinus syndrome clinically. Furthermore, our investigation at the acute isolated sinoatrial node cell level and ion channel level demonstrates convincing evidence of RDV’s specific effects on sinoatrial node cells and channels.

The study by Szendrey et al. (2021) demonstrates that in stably transfected HEK293 cells, RDV not only lacks an effect on the hERG current but also does not block the KCNQ1 + KCNE1 current. However, until recently, Al-Moubarak et al. (2021) demonstrated, contrary to Szendrey et al., that RDV indeed blocks the hERG current in HEK293 transfected cells. This result is consistent with our patch clamp results, showing significant inhibition of hERG current by RDV, which may account for as a major cause the QT interval prolongation and a contributing factor to bradycardia. However, based on the *in vivo* telemetry electrocardiography results and *ex vivo* electrocardiography results, pathological sick sinus syndrome appears to be the main cause of bradycardia. Therefore, close attention should be paid to the cardiac side effects of RDV in clinical use, and appropriate ion channel modulation can be employed to prevent the occurrence of severe sick sinus syndrome.

### 4.1 Limitations

This study primarily investigated the effects of RDV on sinoatrial node cells at the cellular level, focusing on pacemaking and the I_f_ current of the key pacemaking channel HCN4. However, the action potential generation of sinoatrial node cells involves not only the phase 4 primary hyperpolarization-activated current I_f_, but also currents from Nav1.5 channels, T-type and L-type Ca^2+^ channels, as well as the repolarization phase I_Ks_ current (Donald and Lakatta, 2023; Fan et al., 2023). Therefore, effects of RDV on other ion channel currents warrants further investigations. Additionally, unlike typical COVID-19 patients treated with RDV, this study used healthy guinea pigs, which cannot fully reflect the complexity of RDV use in critically ill COVID-19 patients. Although this could be addressed with an *in vivo* COVID-19 guinea pig model, it is currently challenging due to safety considerations and the absence of relevant guinea pig experimental models. Nevertheless, the findings from this study contribute to understanding the cardiac safety profile of RDV when used alone, providing foundational mechanistic insights that could guide future clinical use of RDV and aid in mitigating cardiac adverse reactions associated with its clinical application.

## Data Availability

The original contributions presented in the study are included in the article/Supplementary Material, further inquiries can be directed to the corresponding author.
