# Supplementary material for: Electrophysiological and sick sinus syndrome effects of Remdesivir challenge in guinea-pig hearts
Source: Front Physiol. 2024 Aug 13;15:1436727. doi: 10.3389/fphys.2024.1436727 (PMC11347342; doi:10.3389/fphys.2024.1436727)
Supplement: Supplementary file 1 [file Table1.DOCX]

**Supplementary Table 1.**

Effect of RDV on heart rate in isolated Langendorff-perfused guinea pig hearts

| **Group** | Control | 0.1 μM | 0.3 μM | 1 μM | 3 μM RDV | 10 μM RDV | Wash-out |
| --- | --- | --- | --- | --- | --- | --- | --- |
| **HR (BPM)** | 249.88 ± 11.66 | 243.91 ± 26.90 | 238.02 ± 21.41 | 233.33 ± 16.64 | 220.80 ± 9.41** | 205.01 ± 6.21*** | 230.64 ± 8.66* |

Values are means ± SD in BPM. p value is calculated by homoscedastic Student’s t-test, *P < 0.05, **P < 0.01, ***P < 0.001, n=5.
